# Functional and vascular neuroimaging in maritime pilots with long-term sleep disruption

**DOI:** 10.1007/s11357-024-01417-4

**Published:** 2024-11-12

**Authors:** Lara J. Mentink, Matthias J. P. van Osch, Leanne J. Bakker, Marcel G. M. Olde Rikkert, Christian F. Beckmann, Jurgen A. H. R. Claassen, Koen V. Haak

**Affiliations:** 1 Department of Geriatrics, Radboudumc Alzheimer Centre, Radboud University Medical Center, Nijmegen, The Netherlands; 2 Donders Institute for Brain, Cognition and Behaviour, Radboud University Medical Center, Nijmegen, The Netherlands; 3 Department of Cognitive Science and Artificial Intelligence, School of Humanity and Digital Sciences, Tilburg University, Tilburg, The Netherlands; 4 Department of Radiology, Leiden University Medical Center, Leiden, The Netherlands; 5 Centre for Functional MRI of the Brain (FMRIB), Nuffield Department of Clinical Neurosciences, Wellcome Centre for Integrative Neuroimaging, University of Oxford, Oxford, UK; 6 Department of Cardiovascular Sciences, University of Leicester, Leicester, UK

**Keywords:** Sleep disruption, Maritime pilots, Shift work, Functional MRI, ASL, Vascular damage

## Abstract

The mechanism underlying the possible causal association between long-term sleep disruption and Alzheimer’s disease remains unclear Musiek et al. 2015. A hypothesised pathway through increased brain amyloid load was not confirmed in previous work in our cohort of maritime pilots with long-term work-related sleep disruption Thomas et al. Alzheimer’s Res Ther 2020;12:101. Here, using functional MRI, T2-FLAIR, and arterial spin labeling MRI scans, we explored alternative neuroimaging biomarkers related to both sleep disruption and AD: resting-state network co-activation and between-network connectivity of the default mode network (DMN), salience network (SAL) and frontoparietal network (FPN), vascular damage and cerebral blood flow (CBF). We acquired data of 16 maritime pilots (56 ± 2.3 years old) with work-related long-term sleep disruption (23 ± 4.8 working years) and 16 healthy controls (59 ± 3.3 years old), with normal sleep patterns (Pittsburgh Sleep Quality Index ≤ 5). Maritime pilots did not show altered co-activation in either the DMN, FPN, or SAL and no differences in between-network connectivity. We did not detect increased markers of vascular damage in maritime pilots, and additionally, maritime pilots did not show altered CBF-patterns compared to healthy controls. In summary, maritime pilots with long-term sleep disruption did not show neuroimaging markers indicative of preclinical AD compared to healthy controls. These findings do not resemble those of short-term sleep deprivation studies. This could be due to resiliency to sleep disruption or selection bias, as participants have already been exposed to and were able to deal with sleep disruption for multiple years, or to compensatory mechanisms Mentink et al. PLoS ONE. 2021;15(12):e0237622. This suggests the relationship between sleep disruption and AD is not as strong as previously implied in studies on short-term sleep deprivation, which would be beneficial for all shift workers suffering from work-related sleep disruptions.

## Introduction

Sleep disturbances in midlife have been associated with an increased risk of late-onset Alzheimer’s disease (AD), yet the mechanism linking sleep to AD remains unclear [1–5]. Moreover, the relationship between AD and sleep is thought to be bidirectional [6]. Sleep–wake disturbances are a common symptom of moderate AD [7], but were found to already emerge in the preclinical stages of the disease [8]. Therefore, the association between sleep deprivation and AD could be due to causation as well as reverse causation.

Accumulation of the amyloid beta protein (Aβ) in the brain, a pathological hallmark associated with AD, has been hypothesized to be a possible underlying mechanism [6, 9]. In view of the circadian rhythm of Aβ production and clearance, increased levels of Aβ after sleep deprivation can be explained in two ways. Firstly, increased production of Aβ could be due to higher neuronal and synaptic activity during prolonged wakefulness [6, 10, 11]. Secondly, Aβ may accumulate as a result of decreased clearance of Aβ due to reduced total (deep) sleep time, as the glymphatic system for clearing toxins from the central nervous system is mainly active during sleep [12–14]. In support of this hypothesis, early animal studies indicated an increase in cortical Aβ deposition after sleep deprivation [15–18]. This was translated to humans, where the overnight reduction in Aβ levels in cerebrospinal fluid (CSF) after one night of normal sleep was absent after one night of full sleep deprivation. This was confirmed after one night of selective slow wave sleep disruption [19, 20]. In addition, Winer et al. (2020) observed that reduced slow wave sleep and sleep efficiency were associated with the rate of cortical Aβ accumulation [21]. Taken together, these studies provide some evidence for a causal link between sleep deprivation and AD via the Aβ cascade hypothesis. However, these studies remain limited to short-term effects of sleep on Aβ, and information on longer term effects or on development of AD is lacking.

In our “Sleep-Cognition Hypothesis In Maritime Pilots (SCHIP)” study, in which we investigated a unique group of older maritime pilots, no evidence for increased cortical Aβ accumulation has been found after long-term (approximately 20 years) intermittent sleep disruption, with reduced and fragmented sleep, related to their highly irregular shift work [22]. This finding was unexpected, in view of our hypothesis that the short-term increases in Aβ after sleep disruption, when repeated many times over the years, would result in chronic cortical Aβ accumulation [19, 20]. This therefore challenges the hypothesis that increased Aβ accumulation is the underlying cause of the association between sleep disruption and the increased risk for AD. This is in line with a recent study in mice where chronic sleep deprivation activated metabolic processes in the brain, independent of Aβ accumulation [23]. Therefore, in order to advance our understanding of associations between sleep and neuroimaging markers, we here look more broadly across sets of imaging-derived phenotypes and their association with sleep disruption.

Decreased co-activation of the default mode network (DMN) has been observed in people with AD, and might be present prior to or independent of possible amyloid deposition [24, 25]. The DMN is one of the major resting state networks, showing synchronous activity during rest from spatially distinct brain regions, measurable with functional MRI (fMRI) [26, 27]. The co-activation of the DMN decreases throughout the aging process, with accelerated deactivation in mild cognitive impairment (MCI) and AD [25, 28, 29]. It encompasses also the first regions to show accumulation of Aβ plaques, whereas co-activation is already affected before the Aβ accumulation is measurable with PET [30]. Interestingly, sleep disturbances also directly influence the DMN; one night of total sleep deprivation has been shown to significantly decrease DMN connectivity in healthy, young participants [31]. While the DMN is the most investigated network in the existing literature, a few studies indicate that other resting-state networks may also be affected in sleep deprivation, aging, and AD, i.e. the salience (SAL) and frontoparietal (FPN) networks [32–35]. Therefore, we will extend this study on DMN with an exploratory analysis of the functional co-activation of the SAL and FPN networks and their inter-network connectivity.

Besides resting-state fMRI biomarkers, sleep disruption is potentially also linked with AD through cardiovascular pathways. Although this relation between sleep disruption and AD has not been widely studied, there are indications that disturbed circadian rhythms, short sleep duration, and sleep quality are associated with cerebral small vessel disease and have other cardiovascular consequences such as reduced cerebral perfusion and increased white matter hyperintensities, although the directionality of these associations is not always clear [36–40]. Moreover, perfusion changes have been found very early in the disease cascade of AD [41]. Therefore, an additional aim of our study was to investigate vascular damage and cerebral flood flow (CBF). By looking beyond the amyloid hypothesis, the SCHIP study allows us insight into the possible mechanisms with which sleep disruptions could lead to AD.

## Methods

### Participants

Maritime pilots who previously participated in the SCHIP study [22] were approached to participate in this follow-up study. These pilots guide large international ships from open sea into their final docking position within Dutch harbors. This role does require not only expert technical and navigational skills, but also high physical fitness to board the moving ships on open sea. In addition, their profession (median years in profession = 24 years) causes these participants to have externally induced sleep disruptions. Maritime pilots have a 7-day workweek during which they are on call for 24 h a day, therefore leading to irregular, unpredictable working hours and multiple, fragmented sleep sessions per day. A workweek is followed by a week off with unrestricted sleep. A detailed overview of their sleep schedule, measured using wearable EEG, is available in Mentink et al. [42]. In summary, maritime pilots show fragmented sleep during their workweek, adding up to a total sleep time similar to their week off, but over multiple sessions. In addition, the relative amount of deep sleep increases towards the end of their workweek. In the SCHIP study, while showing a poor subjective sleep quality during their workweeks (mean PSQI = 8.8 ± 2.9), maritime pilots were found to have no intrinsic sleep disorders, normal cognitive function, and no elevated cortical Aβ levels. For more details on this unique population, see Thomas et al. [22, 43]. From the original study population (*n* = 19), 16 maritime pilots (median age = 56 years, age range = 52–61 years) agreed to participate in this follow-up study (Table 1). In addition, we recruited 16 healthy male controls participants, aiming to match the age range of 50–60 years, (median age controls = 59 years, age range = 51 – 66 years), higher education level, and level of physical activity as close as possible. This combination in control participants was exceptionally hard to recruit, leading to some statistically significant demographic differences, even though control participants are within a similar age range and all controls completed higher education with an ISCED score 6 (bachelor or equivalent) or higher. The control participants did not perform shift work and had normal sleep, confirmed by a Pittsburgh Sleep Quality Index of ≤ 5 for the previous month, and regular sleep/wake schedules. Exclusion criteria were use of psychoactive medication, consuming > 30 alcoholic beverages per week, BMI > 30 kg/m^2^, and diagnosed with an intrinsic sleeping disorder. The sample size of our follow-up study was limited by the original study, which was determined with a power calculation aimed to detect differences in amyloid PET-CT imaging [43]. Nevertheless, prior MRI studies with similar sample sizes have detected statistically significant differences in DMN co-activation between people with AD and controls and between different age groups [29, 34, 44]. Therefore, if the influences of long-term sleep deprivation are of a similar, clinically relevant effect size, we expected to detect those even with a limited sample size. The SCHIP study was approved by the institutional review board (IRB) (CMO Region Arnhem-Nijmegen, NL55712.091.16; file number 2016–2337) and was performed in accordance with Good Clinical Practice (GCP) guidelines. We obtained written informed consent from all participants.

**Table 1:** Baseline characteristics of the maritime pilots and healthy controls participating in this SCHIP study follow-up

	Maritime pilots (*N* = 16)	Healthy controls (*N* = 16)	*p*-value
Age, years	56 (54 – 59)	59 (57 – 60.5)	0.04
Sex, males (%)	16 (100)	16 (100)	1
Education, ISCED score	7 (7—7)	6.5 (6—7)	0.008
BMI, kg/m^2^	26.6 (22.7 – 27.0)	25.3 (23.7 – 28.1)	0.95
Amount of working years as maritime pilots	24 (19.5 – 27)	N.A	N.A
Retired at start of current study, *n* (%)	5 (31)	0 (0)	0.018
History of high cholesterol, *n* (%)	1 (6)	1 (6)	1
History of high blood pressure, *n* (%)	1 (6)	2 (13)	0.58
Physical activity > 30 min a day, *n* (%)	14 (88)	15 (94)	0.58
*Once a week, n (%)*	2 (13)	1 (6)	0.58
*Twice a week, n (%)*	4 (25)	2 (13)	0.39
≥ *Three times a week, n (%)*	8 (50)	12 (75)	0.16
Medication use, *n* (%)	3 (19)	5 (31)	0.44
*Antihypertensives, n (%)*	0 (0)	2 (13)	0.16
*Statins, n(%)*	0 (0)	1 (6)	0.35
*Other, n(%)*	3 (19)	4 (25)	0.69
Smoking, *n* (%)	1 (6)	2 (13)	0.58
Alcohol use, number of drinks per week	3 (2 – 6.5)	2 (1 – 5.5)	0.38
Hours since last shift, non-retired maritime pilots only (*N* = 11)	73.5 (69—143)	N.A	N.A

Data displayed as median (IQR) unless otherwise stated. Group differences were tested using a Mann–Whitney *U* test, for non-normally distributed data.

*Abbreviations*: *PSQI*, Pittsburgh Sleep Quality Index; *BMI*, body mass index; *ISCED*, International Standard Classification of Education; *SD*, standard deviation; *IQR*, interquartile range.

### Neuroimaging protocol and data pre-processing

The participants were scanned on a 3T MR scanner (Prisma, Siemens, Germany) at the Donders Centre for Cognitive Neuroimaging. Maritime pilots were scanned during their week off, with a median of approximately 3 days (73.5 h) after their last shift. The MR protocol entailed the acquisition of structural T1-weighted MRI, resting-state fMRI, T2-weighted FLAIR, and arterial spin labeling (ASL) perfusion scans.

#### Resting-state fMRI

Resting-state data were acquired using a 10-min multiband accelerated EPI sequence (TR = 1000 ms, TE = 34 ms, flip angle = 60°, spatial resolution = 2.0 mm isotropic, number of slices = 66, multiband factor = 6). Additionally, we acquired spin-echo EPI data with reversed phase-encode blips for distortion correction purposes (TR = 5186 ms, TE = 47.40 ms, flip angle = 90°, spatial resolution = 2.0 mm isotropic).

Fieldmaps were created using FSL’s topup. Data was collected with reversed phase-encode blips, resulting in pairs of images with distortions going in opposite directions. From these pairs, the susceptibility-induced off-resonance field was estimated using a method similar to that described in [45] as implemented in FSL [46] and the two images were combined into a single corrected one.

Preprocessing of the resting-state fMRI data was performed in FSL’s FEAT. This included removal of the first five volumes, field map correction, realignment to the middle volume using MCFLIRT [47], global intensity normalisation, and 6-mm FWHM spatial smoothing. We then used ICA-AROMA, a data-driven ICA-based method to remove signal components related to head motion [48]. Afterwards, nuisance regression was performed to the white matter and cerebrospinal fluid (CSF) signal. Lastly, we applied a high-pass temporal filter with cut-off frequency of 0.01 Hz and registered the data to 2-mm MNI152 space.

#### 3D FLAIR

Data were acquired using a 3D FLAIR sequence with an acquisition time of 5 min 12 s (TR = 5000 ms, TE = 397 ms, TI = 1800 ms, spatial resolution = 0.5 × 0.5 × 1 mm, GRAPPA acceleration factor = 3).

The MRI T2-FLAIR scans were used to visually score periventricular lesions (PVL), white matter hyperintensities (WMH), lacunes (LAC), and perivascular spaces (PVS). Assessors (LM and LB) were first trained in scoring by an experienced assessor (JC). Sufficient training was confirmed by JC after scoring a practice dataset with T2-FLAIR scans collected at the department of Geriatrics. For scoring the severity of WMH, the Fazekas scale [49] was used. The Fazekas scale makes a distinction between PVL and DWMH, and ranges between 0 and 3. For PVL, 1 was scored in case of caps and a pencil-thin lining (< 5 mm), 2 was scored in case of a smooth halo (> 5 mm), and 3 was scored in case of an irregular cap extending into deep white matter. For DWMH, 0 was scored in case of no MRI-visible DWMH, 1 in case of single lesions of ≤ 9 mm or grouped lesions of < 20 mm, 2 in case of single lesions between 10 and 20 mm or grouped lesions of > 20 mm with no more than connecting bridges between individual lesions, and 3 was scored in case of single lesions or confluent areas of ≥ 20 mm. The number of lacunes (> 3 mm) was counted and enlarged PVS were assessed using a semi-quantitative scale ranging from 0 up to 4, whereby a score 0 is given in case of no MRI-visible PVS, 1 in case of < 10 PVS, 2 in case of 10 to 20 PVS, 3 in case of 21 to 40 PVS, and 4 in case of > 40 PVS [50, 51]. Table 2 lists the cut-off points to score PVL, DWMH, and PVS. In case of disagreement in scoring between assessors, a third researcher (JC) would score the item.

**Table 2:** Scoring matrix for periventricular lesions, deep white matter hyperintensities, and perivascular spaces

**Item ↓**	Score →				
	0	1	2	3	4
PVL	No PVL	Caps and/or pencil-thin lining (< 5 mm)	Smooth halo (> 5 mm)	Irregulars caps extending into deep white matter	-
DWMH	No DWMH	Single lesions ≤ 9 mm or grouped lesions of ≤ 20 mm	Single lesions ≥ 10 mm and < 20 mm or grouped lesions > 20 mm with no more than connecting bridges between individual lesions	Single lesions or confluent areas of ≥ 20 mm	-
PVS	No PVS	< 10	10–20	21–40	> 40

*PVL*, periventricular lesions; *DWMH*, deep white matter hyperintensities; *PVS*, perivascular spaces.

#### Arterial spin labeling

ASL data were acquired using the Siemens standard pulsed ASL FAIR-QUIPSSII sequence (six measurements, single-TI of 1990 ms, bolus duration = 700 ms), with a 3D readout (TR = 4600 ms, TE = 16.28 ms, spatial resolution = 3 mm isotropic, interpolated to 1.5 × 1.5 × 3 mm, 40 slices, echo planar imaging factor = 21).

With FSL’s MCFLIRT, we registered the ASL measurements to the middle volume. Using FSL’s asl file, we then calculated the difference data by subtracting the tag-control pairs, as well as calculated a mean control image to use as a substitute for the M0 image. To calculate a measure of cerebral blood flow (CBF), we used the formula recommended by the ISMRM perfusion study group and the European consortium for ASL in dementia (formula 1 and 2) [52], but due to the absence of a proper M0, further analysis focuses on relative changes in CBF by including the participant’s average whole-brain GM CBF values as covariates in the statistical analysis.

To determine region-specific CBF-values, we selected the hippocampi and posterior cingulate cortex (PCC) from the HarvardOxford cortical and subcortical 2-mm Atlas and registered these ROIs from standard to native space with FSL’s FMRIB’s Linear Image Registration Tool (FLIRT) and FMRIB’s Non-linear Image Registration Tool (FNIRT) [47, 53]. We segmented the T1 image with FSL’s FAST and then similarly to above registered the segmented grey matter to native space [54]. By combining the binarized ROIs with the binarized grey matter mask, we selected only the grey matter voxels within our ROIs. We then utilized FSL’s fslstats to calculate the average value of CBF of the grey matter inside these ROIs. Additionally, we calculated the average value of CBF in the whole-brain grey matter.

For the voxel-wise analysis, we dilated the whole-brain mask output from FAST and registered this mask to native space to create a brain-extracted CBF image. We then registered the CBF images to standard space (MNI152) using FLIRT and FNIRT [47, 53].

### Statistical analyses

#### Resting-state fMRI

A group-level independent component analysis, implemented in FSL’s Multivariate Exploratory Linear Optimised Decomposition into Independent Components (MELODIC), identified 25 resting-state networks [55]. Using dual regression, the subject-specific spatial maps and associated timeseries were estimated [56, 57]. The spatial maps representing the DMN, SAL, and FPN were selected based on visual inspection of the group ICA results.

The between-group differences were assessed using FSL’s Randomise permutation-testing tool (with TFCE inference, 10,000 permutations), with a Bonferroni-corrected *p*-value threshold of 0.05/6 = 0.008 as two contrasts were assessed in each of the three networks [58]. Additionally, we added covariates of age, and, within the group of maritime pilots, history of work-years and, for non-retired maritime pilots, the duration between last shift and MRI scan (representing any short-term sleep deprivation).

Four of the resulting 25 networks showed clear artefacts and were excluded from further analysis. As a quality check, we assessed the group means of all remaining ICA networks (21 of 25 networks) to indicate whether the mean response of each network is different from 0.

In addition to assessing the between-group co-activation of the three RSNs, we were also interested if the correlation of the timeseries between the RSNs differed between groups, i.e. whether there is a between-group difference in connection strength between the RSN’s. With FSLNets, we were able to calculate the full correlation matrix between the subject-specific timeseries of each RSN, the stage 1 dual regression output. Between-group differences were assessed using FSL’s Randomise with 10,000 permutations and corrected for the multiple comparisons across all edges [58].

#### 3D FLAIR

Normality in the lesion scores was tested with a Shapiro–Wilk test. Between-group differences in vascular lesions were assessed using a Mann–Whitney *U* test in MATLAB 2022b (MA, USA).

#### Arterial spin labeling

The average CBF values in the ROIs were non-normally distributed. Therefore, non-parametric permutation tests (FSL’s permutation analysis of linear models (PALM) with 10.000 permutations) were employed to assess group differences in CBF [58]. Average CBF in whole-brain grey matter and summed probabilities of grey matter in the ROIs for partial volume correction were added as covariates to the model.

For the voxel-wise analyses, we also tested for group differences using non-parametric permutation tests (FSL’s Randomise, 10,000 permutations, with TFCE inference) [58]; this time using the whole-brain CBF images in standard space as input. Average CBF in whole-brain grey matter was added as a covariate to the model.

## Results

To study whether maritime pilots with long-term sleep disruption show altered brain biomarkers related to sleep and AD, we compared the 16 maritime pilots with 16 healthy matched controls.

### Resting-state fMRI

**Fig. 1:**
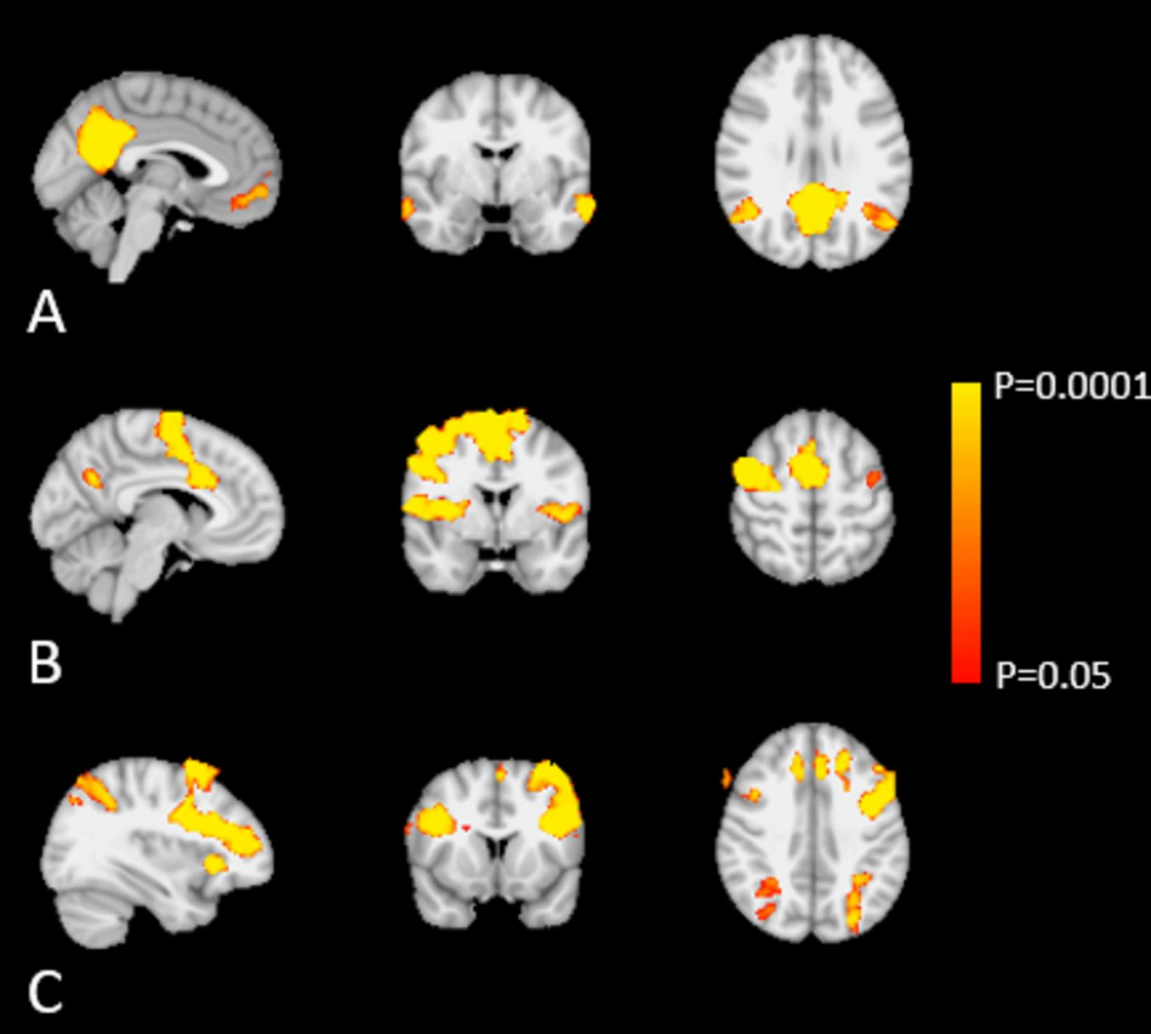
The mean co-activation of the default-mode (A), salience (B), and frontoparietal (C) networks

For the main analyses, i.e. the resting-state fMRI, we studied the hypothesis that maritime pilots show altered RSN co-activation compared to controls; more specifically, we hypothesized altered DMN, SAL, and FPN co-activation. Using group-ICA and dual regression, we were able to identify these networks in each subject and study between-group differences using permutation analyses. Our quality check indicated that the mean co-activation of the 21 meaningful networks from the 25 components was different from 0 (*p* > 0.0001 for all 21 networks in both groups) (Fig. 1).

No significant differences in DMN (pilots > controls: all *p* > 0.28 and t < 5.9; controls > pilots: all *p* > 0.59 and *t* < 5.0), SAL (pilots > controls: all *p* > 0.24 and* t* < 4.8; controls > pilots: all *p* > 0.51 and *t* < 4.9), and FPN (pilots > controls: all *p* > 0.71 and *t* < 4.9; controls > pilots: all *p* > 0.46 and *t* < 5.0) co-activation were detected. Including covariates of age, number of work-years, and short-term sleep deprivation did not alter these results.

Additionally, we were interested in whether there were between-group differences in the connection strength between the networks. Therefore, we tested the full correlation matrix using the subject-specific time courses of these networks. Our analysis did not reveal any significant differences in the intercorrelation of the three RSNs between the maritime pilots and controls (DMN-SAL: uncorrected *p* > 0.3; DMN-FPN: uncorrected *p* > 0.21; SAL-FPN: uncorrected *p* > 0.29; corrected *p* = 1 for the connection strength between the three networks in both directions).

### 3D FLAIR

The intra-class correlation coefficient (ICC) showed moderate to good agreement between the two assessors (ICC = 0.50 for PVS, ICC = 0.79 for LAC, ICC = 0.86 for DWMH, ICC = 0.87 for PVL). Table 3 shows the scores for the maritime pilots and controls, displayed as median and interquartile range. The vascular scores do not differ between the two groups.

**Table 3:** Vascular scores for maritime pilots and healthy controls, displayed as median (IQR) and* p*-values of between-group differences in vascular scores

Item	Maritime pilots (N = 16)	Healthy controls (N = 16)	*p*-value	Cohen’s *d*
PVL	1 (0.5)	1 (1)	0.72	0.07
DWMH	1 (0)	1 (0)	0.77	0.07
LAC	0 (0)	0 (0)	1	0
PVS	2 (1)	2 (0)	0.44	0.14

*PVL*, periventricular lesions; *DWMH*, deep white matter hyperintensities; *LAC*, lacunes; *PVS*, perivascular spaces.

### Arterial spin labeling

**Fig. 2:**
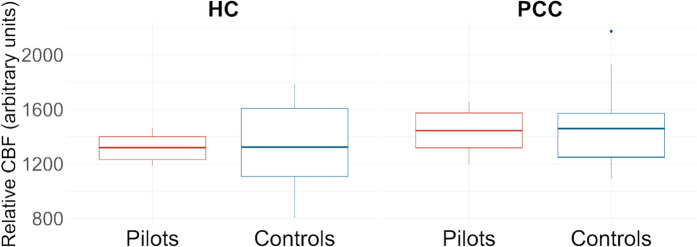
Boxplots indicating the spread in relative CBF in the hippocampi and PCC ROIs between pilots and controls. Abbreviations: CBF, cerebral blood flow; PCC, posterior cingulate cortex; ROI, region of interest

We detected no significant differences in average CBF in the grey matter of the hippocampi and PCC ROIs, corrected for average CBF in whole-brain grey matter and summed probabilities of grey matter in the ROIs for partial volume correction (hippocampus (pilots > controls: *p* = 0.84; controls > pilots: *p* = 0.16; *d* = 0.18), PCC (pilots > controls: *p* = 0.89; controls > pilots: *p* = 0.11; *d* = 0.12)) (Fig. 2).

**Fig. 3:**
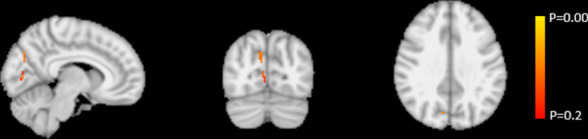
Whole-brain voxel-wise results, showing a small cluster of voxels in the precuneus with higher CBF in maritime pilots compared to controls (uncorrected *p* > 0.043)

In the whole-brain voxel-wise analyses, we found a marginally significant cluster of 3 voxels in the precuneus (corrected *p* > 0.086; *t* < 5.4) in the pilots > controls contrast (Fig. 3).

## Discussion

In this study, we were interested to explore neuroimaging biomarkers related to both sleep disruption and AD. In a previous study in these pilots [22], we found that long-term sleep disruption was not associated with Aβ accumulation, despite previous evidence from studies investigating the effects of short-term sleep deprivation [19, 20]. Therefore, we now aimed to explore other underlying mechanisms that could explain the epidemiological evidence linking long-term sleep disruption to an increased risk for AD. We used a multimodal neuroimaging approach to study putative early brain changes known to be associated with sleep deprivation and/or increased risk for AD [32–35]. Specifically, we were interested in differences in resting-state network co-activation, vascular damage, and cerebral perfusion between participants with long-term sleep disruption, i.e. maritime pilots, and healthy controls.

Using resting-state fMRI, we studied co-activation of the default mode network (DMN), salience network (SAL), and frontoparietal network (FPN). While the DMN is well studied and known to show decreased activity in AD [25, 28], the SAL and FPN are rather understudied in the field of AD. However, these networks might be affected as a previous study has indicated the triple-network model consisting of DMN, SAL, and FPN to be affected in AD and the same networks were affected after short-term sleep deprivation [59, 60]. Our results showed no differences between maritime pilots and healthy controls, in contrast to short-term sleep deprivation studies, where decreased DMN, SAL, and FPN activations were found after acute total sleep deprivation [31, 35, 61]. While multiple studies are available on acute total sleep deprivation, evidence from long-term sleep disruption as assessed here is lacking. Therefore, we cannot be sure whether decreased RSN co-activation is a short-term effect, only occurring after acute total sleep deprivation, or whether it could still be an effect of long-term sleep disruption without any compensatory pauses. Additionally, we were interested whether the connectivity strength between the three RNSs differed between groups; however, no significant differences in between-network connectivity strength were found in our data between maritime pilots and controls. As this has not been studied in relation to sleep disruption, the between-network connectivity strength might only be affected in a later stage of AD, or in sleep disruption without compensatory pauses. We found no indication that it is an early biomarker of the disease.

A few studies have explored the relationship between sleep disruption and cerebrovascular damage due to for example increased oxidative stress and blood–brain-barrier impairment [62]. This could be an alternative underlying mechanism between sleep disruption and AD [63, 64]. Results yielded in previous studies are mixed, showing both increased DWMH and increased small vessel disease in middle-aged and older adults with self-reported short sleep duration [39, 40, 65], as well as no association between short sleep duration at midlife and late-life vascular damage and AD [66]. In line with Lutsey et al. [66], we found no association between sleep disruption and increased vascular damage, as vascular scores in both groups correspond to observations in healthy older adults [51]. However, a direct comparison between studies is impeded due to the differences in sleep disruption between study populations (self-reported short sleep duration vs long-term work-related sleep disruptions). Two studies have investigated disrupted circadian rhythms, more closely resembling the sleep disruption of our study population. These studies both found an association between disturbed 24-h activity rhythms and increased burden of cerebral small vessel disease; however, the directionality of this association could not be confirmed [36, 38]. A confounder in sleep disruption studies might be the (unknown) presence of obstructive sleep apnoea (OSA). Multiple studies in populations with OSA investigated its relationship with AD through vascular damage because people with OSA have higher cardiovascular risk and have increased oxidative stress due to their nocturnal hypoxemia, possibly exacerbating neurovascular damage [66–69], as described in detail in the review by Daulatzai (2015) [70]. Indeed, the meta-analysis by Bubu et al. revealed that among sleep disorders, OSA carries the greatest relative risk (RR) for AD [1].

In our population of maritime pilots with long-term sleep disruptions, we specifically explored CBF in the hippocampus and PCC, as regional CBF changes in areas already affected in early AD are thought to drive global CBF changes [71]. In addition, we investigated whole-brain grey and white matter CBF. We found no differences, compared to normal controls, in the hippocampal and PCC ROIs in the maritime pilots. Our whole-brain voxel-wise analyses showed a small cluster with marginally increased CBF in maritime pilots (uncorrected *p* > 0.043); however, this did not survive correction for multiple testing. Comparison is limited due to the nature of the sleep disruption studied here, as previous work has been limited to acute short-term sleep deprivation experiments. Such studies found decreased cerebral perfusion after total sleep deprivation and sleep restriction in healthy young adults [72, 73].

Several hypotheses could explain our results. Firstly, there may be a true null effect in the causal link between externally induced long-term sleep disruptions and the development of AD. Taking a closer look at the large meta-analysis by Bubu et al. [1], the highest RR for AD was found in OSA, a sleep-related breathing disorder [1]. Furthermore, most of the included studies examined middle-aged and older individuals with a short follow-up. This could suggest that the associations could be driven by a specific sleep disorder (e.g. OSA) or by reverse-causality, wherein the reported sleep disorder is an early symptom of AD. An alternative interpretation of our results could be that a causal link between sleep disruptions and AD does exist but was not detectable in our small sample size. However, our group had a median exposure of 24 years to sleep disruption. Therefore, if sleep disruption is indeed a relevant causal contributor to AD, such a long term and consistent exposure should be expected to show an effect, even in a relatively small sample, especially since we examined sensitive neuroimaging biomarkers of network systems involved in early stages of AD. Thirdly, there may be a causal link between poor sleep and AD, as indicated by studies on acute effects on short-term total sleep deprivation, but compensation for these acute effects is possible; maritime pilots have a week off after each workweek, which may have safeguarded them from long-term sleep deprivation and increased risk for AD. To avoid any confounding factors of intrinsic sleep disorders, we included a population with externally induced sleep disruption. Our participants continued this occupation for a long time, which they might not have done when they would experience significant negative effects from shift work. We therefore may have selected a highly resilient population. Moreover, previous work in this group of maritime pilots illustrates a possible compensation mechanism of increasing total sleep time and the relative amount of deep sleep during their week off [42]. Fourthly, the causal link may be only present in a selected population. Recent research has demonstrated the multifactorial nature of AD, where the emergence of the disease is driven by multiple causal factors; therefore, sleep disruption in itself might not be enough to initiate AD development [74]. In contrast, our participants are healthy and, to our knowledge, have limited other risk factors for AD. An interesting factor to assess would have been APOE ε4-status, as APOE ε4-carriership might modulate the relationship between sleep disruption and AD [75]; however, our ethical review board at the time did not allow individual APOE genotype determination in this study.

For future studies, we would suggest including a larger and more diverse population regarding ancestry, sex, level of education, lifestyle factors, and type of sleep disruption. When studying a population with work-related sleep disruption (i.e. shift work), it is important to carefully define their work and sleep schedules as well as the severity of sleep disruption and quantified (lack of) deep sleep, as research in the Danish Nurse Cohort has shown that not all types of shift work are associated with increased risk for AD [76], impacting generalizability across types of shift work and towards the general population. Furthermore, the field requires more longitudinal studies starting at a younger age compared to the studies in the large meta-analyses [1, 77], to accurately explore whether we are studying a causal relationship as opposed to reverse-causation.

In conclusion, we studied long-term work-related sleep disruption in an interesting group of shift-workers, maritime pilots. We decided to look beyond the amyloid hypothesis by investigating possible alternative underlying mechanisms affecting resting-state networks and cerebrovascular markers, which may present prior to or independent of Aβ accumulation. Here, we did not find evidence of impacted resting-state network co-activation, vascular damage, or cerebral perfusion in our group of maritime pilots compared to healthy controls, which does not support a causal association between this type of sleep disruption and increased risk for AD. As the existing research predominantly concentrates on acute total sleep deprivation, which likely does not represent potential long-term risks, we believe that more longitudinal studies need to be conducted on long-term sleep disruptions to better understand the bidirectional relationship between sleep and AD, along with exploring potential compensation strategies.

## Data Availability

The data that support the findings of this study will be placed in a restricted access data sharing collection (DSC) on the Radboud Data Repository and made available upon request.
