# Correction to: Functional and vascular neuroimaging in maritime pilots with long‑term sleep disruption

**DOI:** 10.1007/s11357-025-01617-6

**Published:** 2025-04-26

**Authors:** Lara J. Mentink, Matthias J. P. van Osch, Leanne J. Bakker, Marcel G. M. Olde Rikkert, Christian F. Beckmann, Jurgen A. H. R. Claassen, Koen V. Haak

**Affiliations:** 1 Department of Geriatrics, Radboudumc Alzheimer Centre, Radboud University Medical Center, Nijmegen, The Netherlands; 2 Donders Institute for Brain, Cognition and Behaviour, Radboud University Medical Center, Nijmegen, The Netherlands; 3 Department of Cognitive Science and Artificial Intelligence, School of Humanity and Digital Sciences, Tilburg University, Tilburg, The Netherlands; 4 Department of Radiology, Leiden University Medical Center, Leiden, The Netherlands; 5 Centre for Functional MRI of the Brain (FMRIB), Nuffield Department of Clinical Neurosciences, Wellcome Centre for Integrative Neuroimaging, University of Oxford, Oxford, UK; 6 Department of Cardiovascular Sciences, University of Leicester, Leicester, UK

**Correction to: GeroScience**

10.1007/s11357-024-01417-4

The original version of this article unfortunately contained errors in the abstract section.

In page 1, 1st and 2nd sentences under the abstract section, the two references Musiek et al. 2015 and Thomas et al. 2020 should be deleted.

The 1st and 2nd sentences should be corrected FROM “The mechanism underlying the possible causal association between long-term sleep disruption and Alzheimer’s disease remains unclear Musiek et al. 2015. A hypothesised pathway through increased brain amyloid load was not confirmed in previous work in our cohort of maritime pilots with long-term work-related sleep disruption Thomas et al. Alzheimer’s Res Ther 2020;12:101.” TO “The mechanism underlying the possible causal association between long-term sleep disruption and Alzheimer’s disease remains unclear. A hypothesised pathway through increased brain amyloid load was not confirmed in previous work in our cohort of maritime pilots with long-term work-related sleep disruption.”

In page 2, second last sentence under the abstract section, reference Mentink et al. should be deleted.

The second last sentence should be corrected FROM “This could be due to resiliency to sleep disruption or selection bias, as participants have already been exposed to and were able to deal with sleep disruption for multiple years, or to compensatory mechanisms Mentink et al. PLoS ONE. 2021;15(12):e0237622.” TO “This could be due to resiliency to sleep disruption or selection bias, as participants have already been exposed to and were able to deal with sleep disruption for multiple years, or to compensatory mechanisms.”

The publisher sincerely apologizes for this error and any inconvenience caused to the authors and readers of the journal.

